# Insertion/deletion-activated frame-shift fluorescence protein is a sensitive reporter for genomic DNA editing

**DOI:** 10.1186/s12864-019-5963-z

**Published:** 2019-07-24

**Authors:** Akhilesh Kumar, Michael D. Birnbaum, Balaji T. Moorthy, Jayanti Singh, Anna Palovcak, Devang M. Patel, Fangliang Zhang

**Affiliations:** 1 Department of Molecular and Cellular Pharmacology, University of Miami, Miller School of Medicine, Miami, FL 33136 USA; 2 Present address: Department of Botany, Banaras Hindu University, Varanasi, 221005 India; 3 Department of Otolaryngology, University of Miami, Miller School of Medicine, Miami, FL 33136 USA; 4 Department of Biochemistry and Molecular Biology, University of Miami, Miller School of Medicine, Miami, FL 33136 USA; 5 Sylvester Comprehensive Cancer Center, University of Miami, Miami, FL 33136 USA

**Keywords:** Insertion-deletion, In-del, Reporter, CRIPSR-Cas9, Genome editing, NHEJ

## Abstract

**Background:**

Reporter methods to quantitatively measure the efficiency and specificity of genome editing tools are important for the development of novel editing techniques and successful applications of available ones. However, the existing methods have major limitations in sensitivity, accuracy, and/or readiness for in vivo applications. Here, we aim to develop a straight-forward method by using nucleotide insertion/deletion resulted from genome editing. In this system, a target sequence with frame-shifting length is inserted after the start codon of a cerulean fluorescence protein (CFP) to inactivate its fluorescence. As such, only a new insertion/deletion event in the target sequence will reactivate the fluorescence. This reporter is therefore termed as “Insertion/deletion-activated frame-shift fluorescence protein”. To increase its traceability, an internal ribosome entry site and a red fluorescence protein mCherryFP are placed downstream of the reporter. The percentage of CFP-positive cells can be quantified by fluorescence measuring devices such as flow cytometer as the readout for genome editing frequency.

**Results:**

To test the background noise level, sensitivity, and quantitative capacity of this new reporter, we applied this approach to examine the efficiency of genome editing of CRISPR/Cas9 on two different targeting sequences and in three different cell lines, in the presence or absence of guide-RNAs with or without efficiency-compromising mutations. We found that the insertion/deletion-activated frame-shift fluorescence protein has very low background signal, can detect low-efficiency genome editing events driven by mutated guideRNAs, and can quantitatively distinguish genome editing by normal or mutated guideRNA. To further test whether the positive editing event detected by this reporter indeed correspond to genuine insertion/deletion on the genome, we enriched the CFP-positive cells to examine their fluorescence under confocal microscope and to analyze the DNA sequence of the reporter in the genome by Sanger sequencing. We found that the positive events captured by this reporter indeed correlates with genuine DNA insertion/deletion in the expected genome location.

**Conclusion:**

The insertion/deletion-activated frame-shift fluorescence protein reporter has very low background, high sensitivity, and is quantitative in nature. It will be able to facilitate the development of new genome editing tools as well as the application of existing tools.

**Electronic supplementary material:**

The online version of this article (10.1186/s12864-019-5963-z) contains supplementary material, which is available to authorized users.

## Background

Genome editing tools have begun to generate revolutionary impacts on the biomedical research field [1]. Through the efforts of many researchers in last two decades, a variety of genome editing tools have been introduced, which include meganucleases [2], zinc finger nucleases (ZFNs) [3], transcription activator-like effector-based nucleases (TALEN) [4], the clustered regularly interspaced short palindromic repeats (CRISPR) and CRISPR-associated genes (Cas) [5, 6]. While these tools utilize different enzymes, genome editing in principle is based on DNA double stranded break (DSB) [1]. Despite the encouraging progress, many limitations still exist for the currently available methods for their efficiencies, specificities, and requirements of specific assisting factor and/or conditions. For example, the current version of CRISPR-Cas has considerable off-target effects and cannot edit mitochondrial genome due to the difficulty of delivering guide RNA (gRNA) to mitochondria. While TALEN can overcome these challenges, finding a suitable target sequence for this tool can be a challenge. To further propel the progress of genome editing for cell modification and eventual therapeutic application, extensive efforts are currently being invested for developing new genome editing tools to achieve higher efficiency, better precision, and wider application conditions. Such tasks will require a convenient method to quantitatively evaluate the efficiency and specificity of genome editing. In addition, the application of currently available genome editing also can benefit from an evaluating tool for optimization. For example, when optimizing the CRISPR-Cas system, several gRNAs targeting different regions would be compared to find one with suitable efficiency and specificity. However, these needs have not been sufficiently met.

The DSB induced by genome editing tools is expected to be followed by either the non-homologous end joining (NHEJ) or homology-directed repair (HDR) inside the cell, which can be used as a surrogate for the detection of genome editing in vivo [7]. However, HDR in principle is a low-frequency event and it requires exogenous donor templates [8, 9], which are expected to introduce additional variations in data. In comparison, NHEJ-based methods are more straight-forward and robust. One of such methods is the Surveyor nuclease mutation detection assay. In this assay, the edited sequence and the original sequence were annealed together in vitro for the cleavage of a mismatch-sensitive endonuclease [10]. This technique has been frequently used to validate DNA editing in isolated cell colonies. However, because saturating PCR is required to generate the DNA fragments for test, this method is not sensitive to low-frequency mismatch and also not suitable for a mixed cell population. In addition, the readout of the Surveyor assay relies on electrophoresis, making it difficult to be adapted for cell-based applications that are essential for many types of high-throughput screenings. The other commonly used method is based on NHEJ-induced inactivation of an enzyme or fluorescence protein, where the inactivation or loss of a protein would indicate a genome editing event [11, 12]. While this method is more adaptable for cell-based assays, it also has several obvious limitations. First, this technique is not suitable to detect low-efficiency genome editing events either, because a minor signal loss can be easily confused as spontaneous signal fluctuation. More importantly, the inactivation/reduction of fluorescence or enzymatic activity cannot distinguish tools for gene editing or gene silencing. The limitation of this method was evident in the case of NgAgo, which was initially considered as a gene editing tool, partly because it was capable of reducing GFP as a test target factor. However, it was later suggested that it is more likely to be a gene silencing enzyme [13–18]. As an inverse logic to the loss of signal, again of a fluorescence signal can be detected with higher sensitivity and accuracy. As such, several attempts have been tried to develop a method where the detection of a fluorescence is directly associated with a genomic editing event. Examples include the “Traffic light reporter” and a lateral derivative [19, 20]. In these methods, a GFP is linked with a mCherryFP by a T2A self-cleaving sequence. A nuclease target sequence is placed in the middle of GFP to disrupt the expression of both fluorescence proteins. With the application of genomic editing system and a donor template containing the coding sequence of GFP, a successful HDR event will result in the recovery of the GFP signal. However, the usage of GFP in this assay appeared to hinder the direct adaptation of this method, because GFP is a popular marker for many vectors carrying the genome editing components. The reporter can also indicate NHEJ event by the reactivation of the downstream mCherryFP signal. However, the detection sensitivity of NHEJ appears to be on the low end (usually at < 2% frequency in the cell population and similar to that of detected HDR events [19, 20], considering that NHEJ usually takes place in a very high frequency after DSB and is known to be more efficient than HDR [21]. Probably, for these reasons, usage of these reporters for the evaluation of genome editing tools are rather uncommon. Therefore, to continue developing new genome editing tools, and to facilitate the application of currently existing tools, it is essential to develop a new method that unambiguously detect genome editing with high sensitivity and is readily compatible for cell-based assays.

In this study, we developed a novel reporter system to evaluate the efficiency of the genome editing. In this system, a target sequence is placed in a multiple cloning site (MCS) containing NotI and XhoI, which is right after the start codon of a fluorescence protein to generate a frame-shift of the open reading frame (ORF). Only when an in/del event following a successful genome editing and NHEJ on the target sequence is expected to reactivate the fluorescence. To facilitate the quantification, an internal ribosome entry site (IRES) and a red fluorescence protein, mCherryFP, is placed after the reporter, As such, the ratio of the two fluorescence can provide quantitative measurement for the efficiency of the genome editing with high accuracy and sensitivity. This in/del-activated fluorescence protein is compatible with cell-based assays, can be used as a powerful tool for the continued development of additional genome editing tools, and to facilitate the design of targeting factors such as gRNA in the currently available tools.

## Results

### Construction of a frame-shift fluorescence protein to report in/del events

**Fig. 1:**
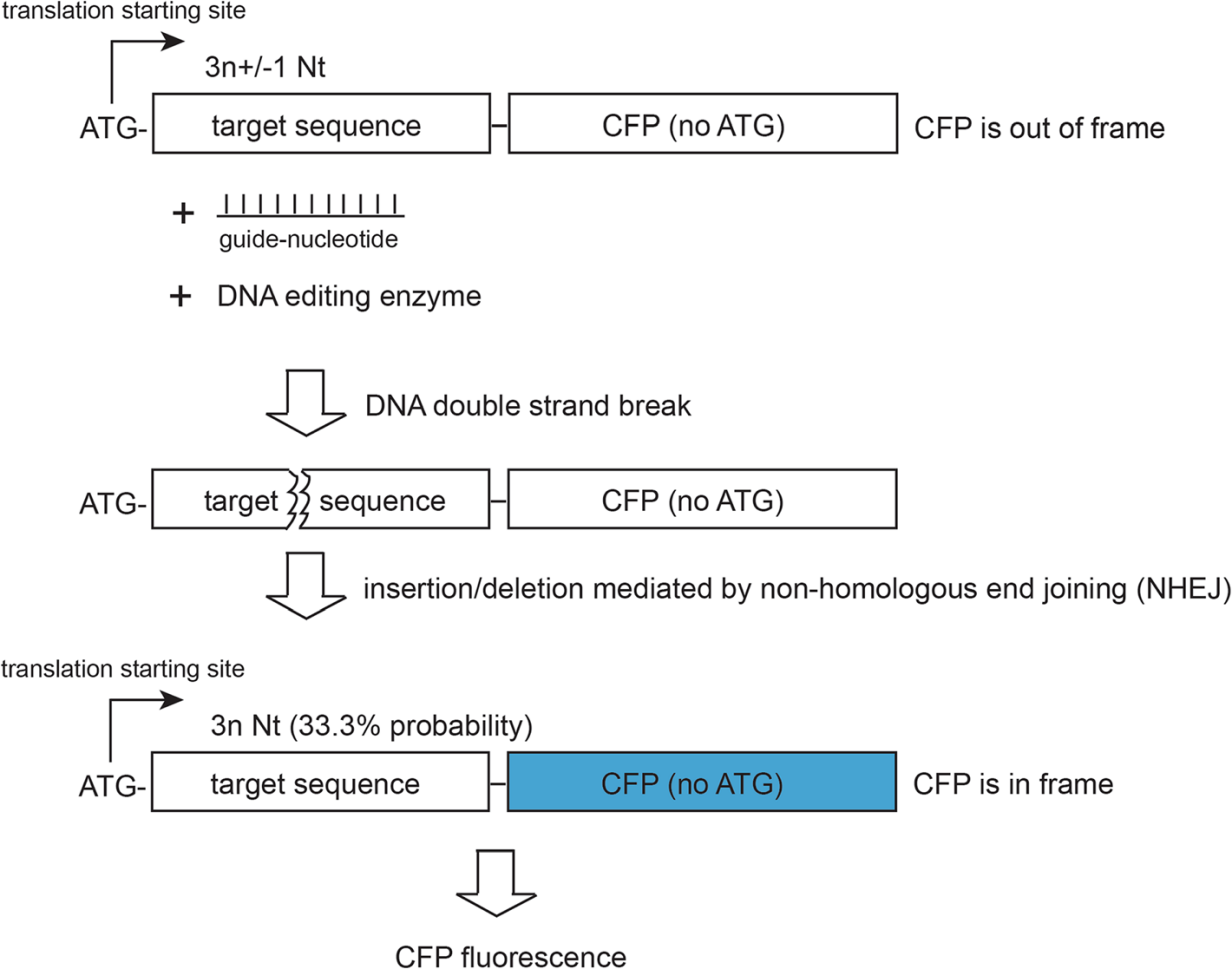
The principle for a frame-shift fluorescence protein that is specifically activated by insertion/deletion (in/del) of nucleotides. See Additional file 1: Figure S1 for the details of the vector structure. As illustrated in the scheme, a target nuleotide sequence with the length of 3n +/− 1 (*n* > =1) is inserted between a start-codon (ATG) and the rest of the coding sequence of a fluorescence protein such as CFP. As such, this fluorescence protein is not expected to be translatable. However, if an in/del event takes place at the inserted target sequence, by a probability of about 1/3, the resulted sequence will become in frame for the translation of the fluoresence protein

To facilitate the efficient transfection and stable insertion of our engineered reporter system in the host genome, we used lentiviral vector for our reporter construction. As a demonstration, we used the coding sequence of cerulean fluorescence protein (CFP) as template for engineering because it contains no in-frame ATG codon in its 5′-region other than the first ATG, thus minimizing the risk of internal translation initiation. The intended target sequence is inserted between the start codon and the rest of the CFP sequence. The length of the inserted sequence is designed to create a frame shift, which, with an optional inclusion of a premature STOP codon, prevents the translation of a functional CFP. As illustrated in Fig. 1, a double-strand break can be created on the target sequence by the genome editing tool and repaired by in/del in NHEJ. Assuming completely random length in spontaneous in/del, there is a 33.3% chance the coding sequence for CFP will become in-frame for the start codon, leading to reactivation of CFP fluorescence. This reporter is termed as frame-shift CFP (FsCFP). To maximize the traceability of the reporter, an internal ribosome entry site (IRES) and mCherryFP, are added in the downstream region (Additional file 1: Figure S1).

### The frame-shift fluorescence reporter is sensitive and quantitative for the detection of genome editing

**Fig. 2:**
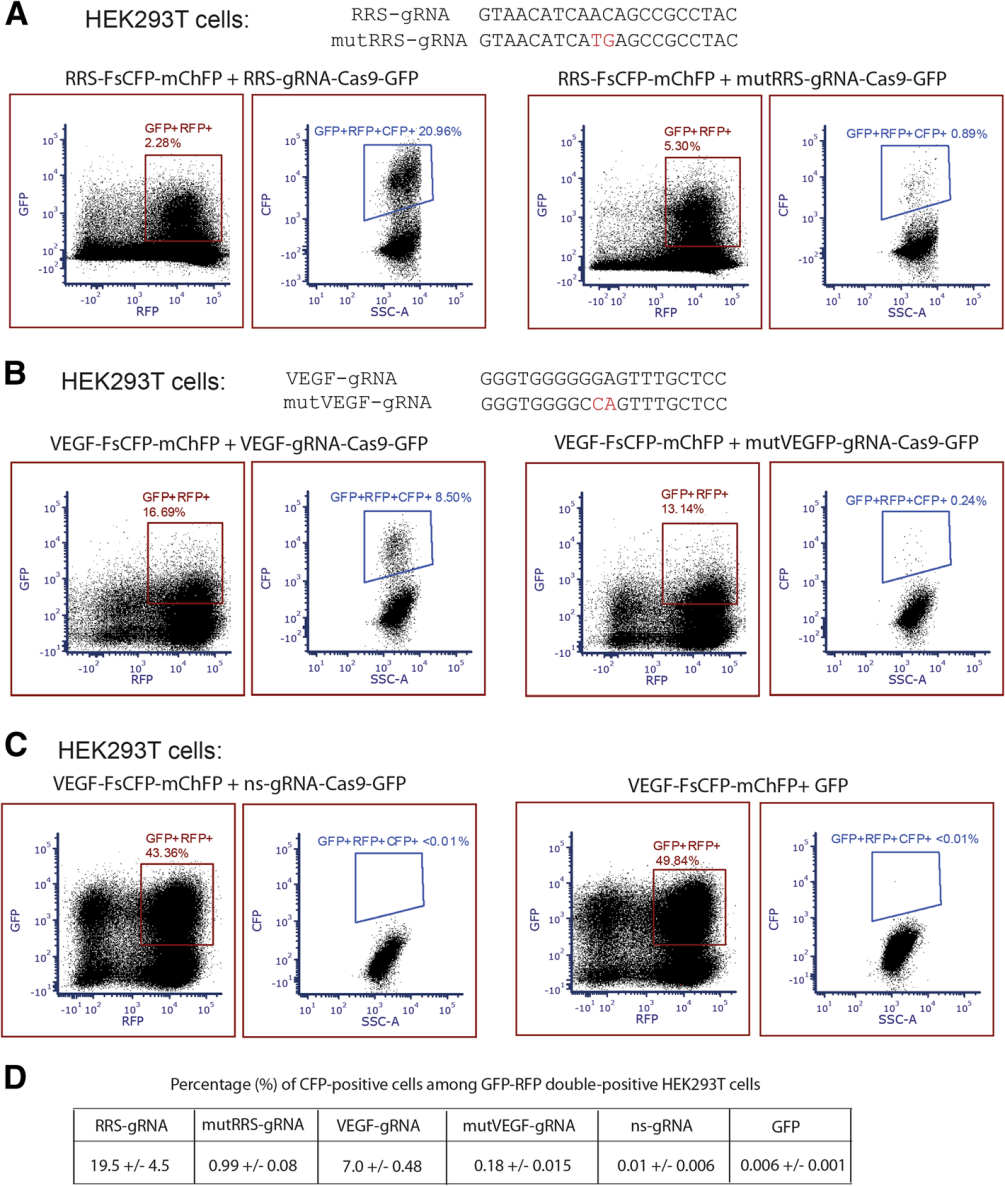
The frame-shift CFP (FsCFP) reporter successfully distinguished genome editing events of different targeting efficiencies, with very low noise signals. See also Additional file 1: Figure S2 and S3 for the establishment of the detection gatings, and S4 for the intensity of background CFP signal. **a** Genome editing was performed on a target sequence derived from the RRS gene of Chinese hamster. The corresponding gRNA is not expected to react with the essential RRS gene in the HEK293T cells, which is the host cell for the editing. The sequence of the wildtype gRNA and the mutant gRNA are presented on top, where the two mutated nucleotides in the center of the mutRRS-gRNA are indicated by red color. On the bottom, two representative flow charts show the HEK293T cells transduced with the FsCFP with the RRS-targeting sequence and the wildtype or mutatnt RRSgRNA-Cas9. Among the cells expressing both the mCherryFP and GFP, which are the markers for the FsCFP and the gRNA-Cas9, repectively, a subset of them also exhibited detectable CFP signals. **b** Similar to A, except that a target sequence derived from human VEGF and the correponding gRNA are used. **c** Representative flow charts of cells with VEGF-FsCFP and either a non-targeting sequence gRNA (ns-gRNA) and Cas9, or just GFP alone without gRNA and Cas9. In both the cases, the ratio of CFP-positive cells was close to or lower than 0.01%. **d** Quantification of CFP-positive events detected in mCherryFP and GFP double positive cells with different gRNAs as shown in **a**, **b** and **c**. The mean value was calculated from independent repeats (*n* = 4 for RRS and mutRRS; *n* = 3 for the others). The error of means were shown after the +/− sign

To construct a targeting vector for the demonstration, a 20 nucleotides N-termianl fragment of the arginyl-tRNA synthase (RRS) gene from Chinese hamster was used as the target sequence in the reporter vector. The reporter vector was stably transduced into human embryonic kidney (HEK) 293 T cells, in which the endogenous human RRS gene is not expected to react with the targeting sequence. To test whether the designed FsCFP can reliably detect genome editing, the gRNA corresponding to the targeting sequence (termed RRS-gRNA) is used. The RRS-gRNA was cloned in a vector carrying Cas9 and a GFP marker, which was then co-transduced with the FsCFP-mCherryFP reporter into the HEK293T cells. The fluorescence of these cells were then analyzed by flow cytometer, in which the detection gating for GFP and mCherryFP was set up with cells that express either only GFP, only mCherryFP, or none (Additional file 1: Figure S3). In the cells transduced with both the reporter and the corresponding gRNA (green and red fluorescence double positive), we found a significant percentage (nearly 20%) of these cells also exhibit CFP signals, indicating successful reporting of in/del events. To test whether this method is sensitive for low-frequency in/del events, two central nucleotides in the gRNA were mutated, which is expected to significantly reduce the targeting efficiency. When this mutated gRNA (mutRRS-gRNA) is used with the reporter, a much reduced yet still consistent CFP signal was detected in the cells with both GFP and mCherryFP (Fig. 2a).

To validate that the above phenomenon is not sequence-specific, we repeated the experiment by using a gRNA sequence previously shown to efficiently target the gene of human vascular endothelial growth factor (VEGF) [22], which is not essential for the growth of HEK 293 T cells. We found that the FsCFP-mCherryFP clearly detected substantial positive events with the VEGF-targeting gRNA, and a much reduced level with the mutant gRNA (Fig. 2b).

To examine potential impacts from background fluorescence or from non-specific events such as spontaneous mutation on the reporter, we tested the effects of either the Cas9-GFP vector carrying a non-specific gRNA (ns-gRNA), or just a vector expressing the GFP alone. We found that the level of the positive events generated by ns-gRNA is at least two order of magnitude lower than a typical wild type gRNA, and at least one order of magnitude lower than a typical mutant gRNA. The positive events detected in the presence of GFP alone (without Cas9 or gRNA) are even lower (Fig. 2c, d). These results indicate that non-specific events or background noise contribute minimal signals to the results.

**Fig. 3:**
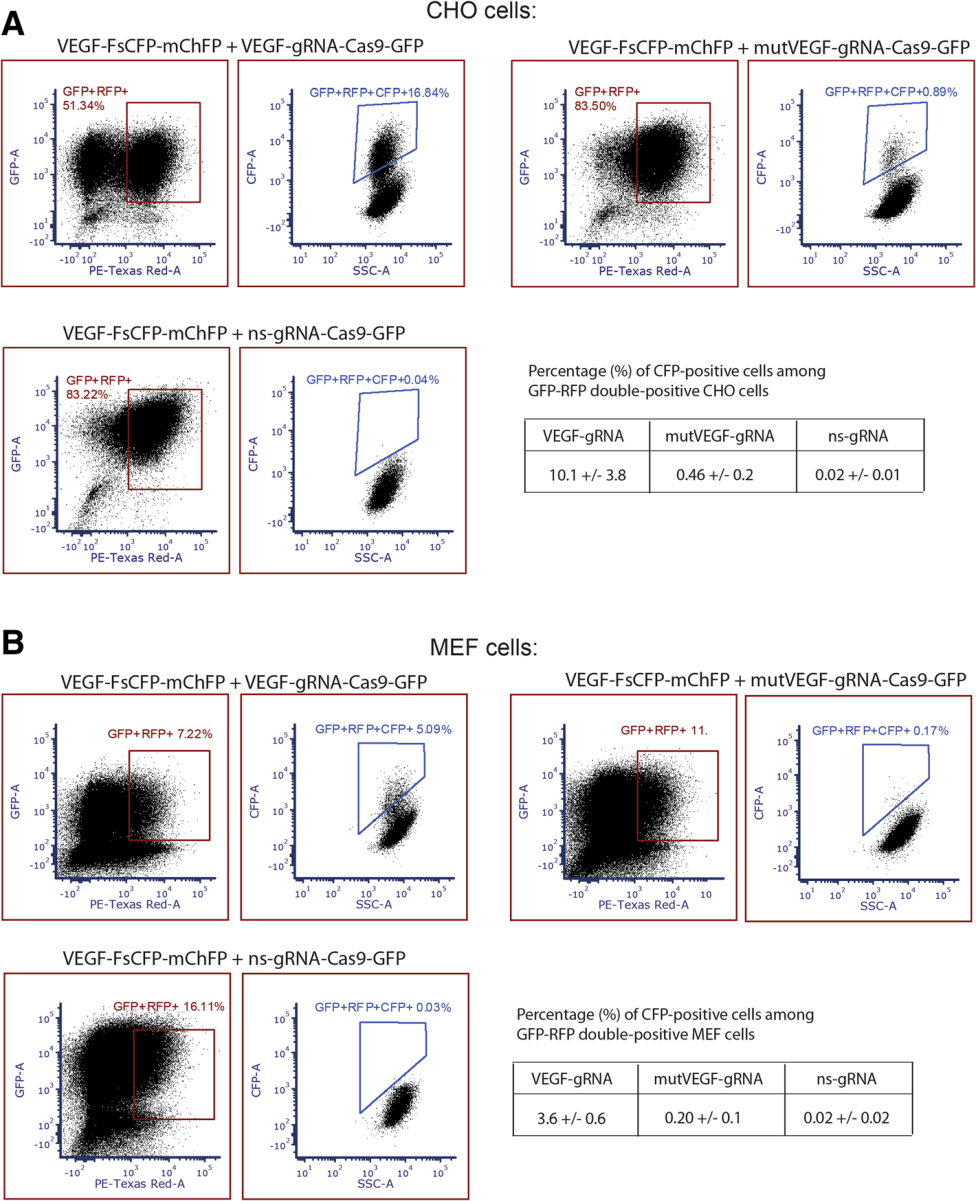
The application of the frame-shift CFP (FsCFP) reporter in two other cell lines also can effectively detect genome editing events with good quantitative quality and low background signals. See Additional file 1: Figure S4 and S5 for the background CFP signal of these cells. **a** The host cell CHO was stably induced with a FsCFP reporter containing target sequence derived from hVEGF. To initiate genome editing, vectors containing Cas9 and either the corresponding gRNA, a mutant gRNA, or a non-specific (ns) gRNA were used, with GFP as a traceable marker, all similar as in Fig. 2b. On the bottom right corner is the quantification of CFP-positive events detected in mCherryFP and GFP double-positive populations. The mean value were calculated from independent repeats (*n* = 3). The error of means were shown. **b** similar to A, except that MEF were used as host cells, and that *n* = 5 for the cells with nsgRNA, *n* = 3 for other cells

To further validate that our reporter is functioning efficiently with different cell lines from different organisms, we used Chinese hamster ovary (CHO) cell and Mouse embryonic fibroblast (MEF) cell. The reporter carrying the VEGF sequence as mentioned above was used as the target. In both cell lines, we found that our reporter could clearly detect and differentiate the positive events in wild type and mutant gRNA, while having minimal background signal when a non-specific gRNA was used (Fig. 3a for CHO, Fig. 3b for MEF, and Additional file 1: Figure S4 and S5).

Therefore, our data show that the designed reporter has sufficient sensitivity to detect low frequency of in/del events driven by genome editing, has the quantitative capacity to distinguish different editing efficiencies, has very low non-specific background signals, and can be used with different cell lines.

### The frame-shift fluorescence reports genuine in/del events

**Fig. 4:**
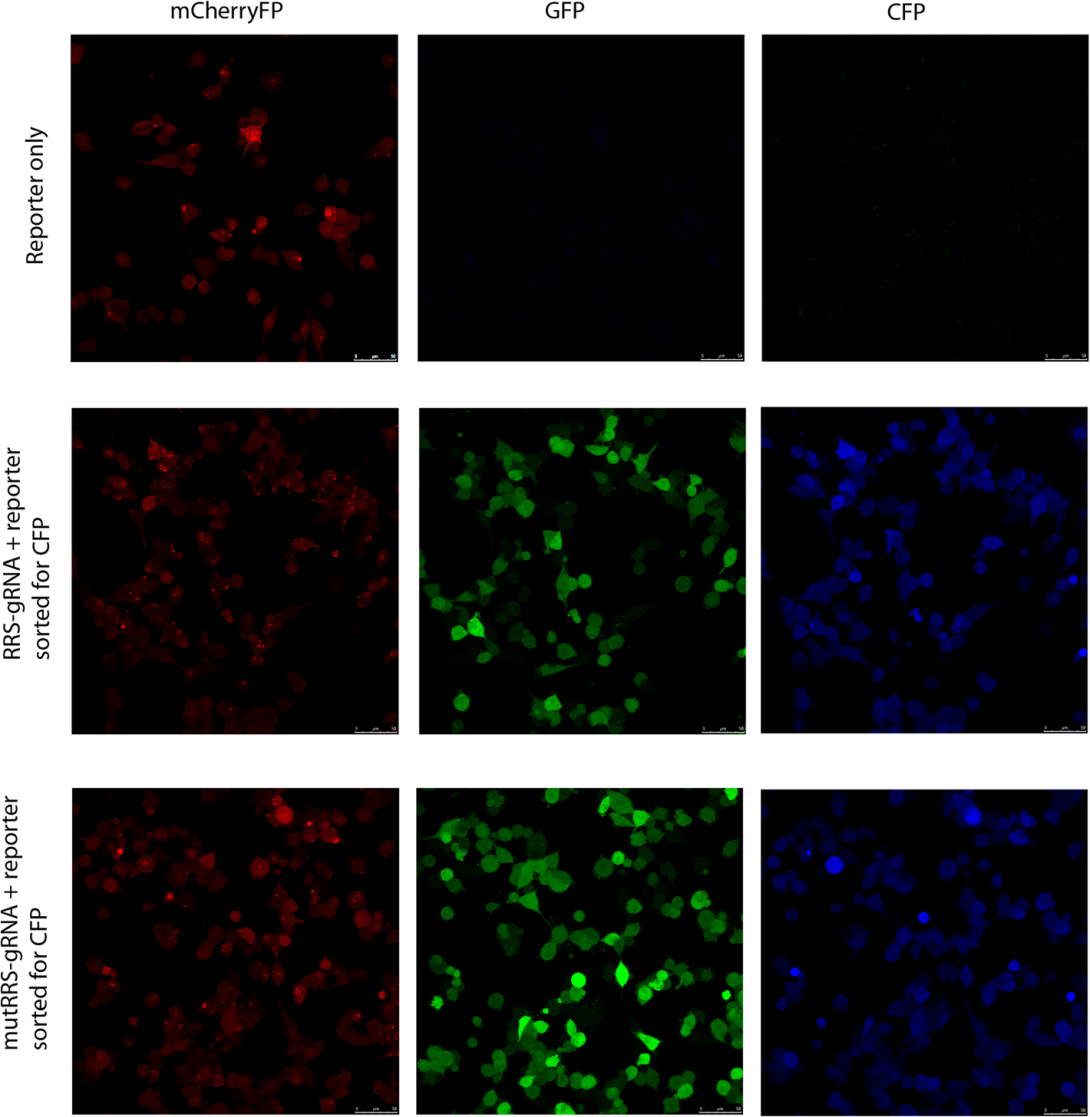
Genuine CFP signals were observed under microscope for the in/del-activated CFP cells enriched by the flowcytometer. HEK293T cells having FsCFP reporter with RRS target sequence and corresponding wildtype or mutant gRNA were sorted for positive CFP signals as decribed in Fig. 2a. The fluorescence of these cells was examined under microscope. Besides the expected GFP and RFP signals, prominent CFP signal was also detected in these cells. As a control, cells carring only the FsCFP reporter don’t exhibit detectable CFP signal. Scale bars indicate 50 μm

**Fig. 5:**
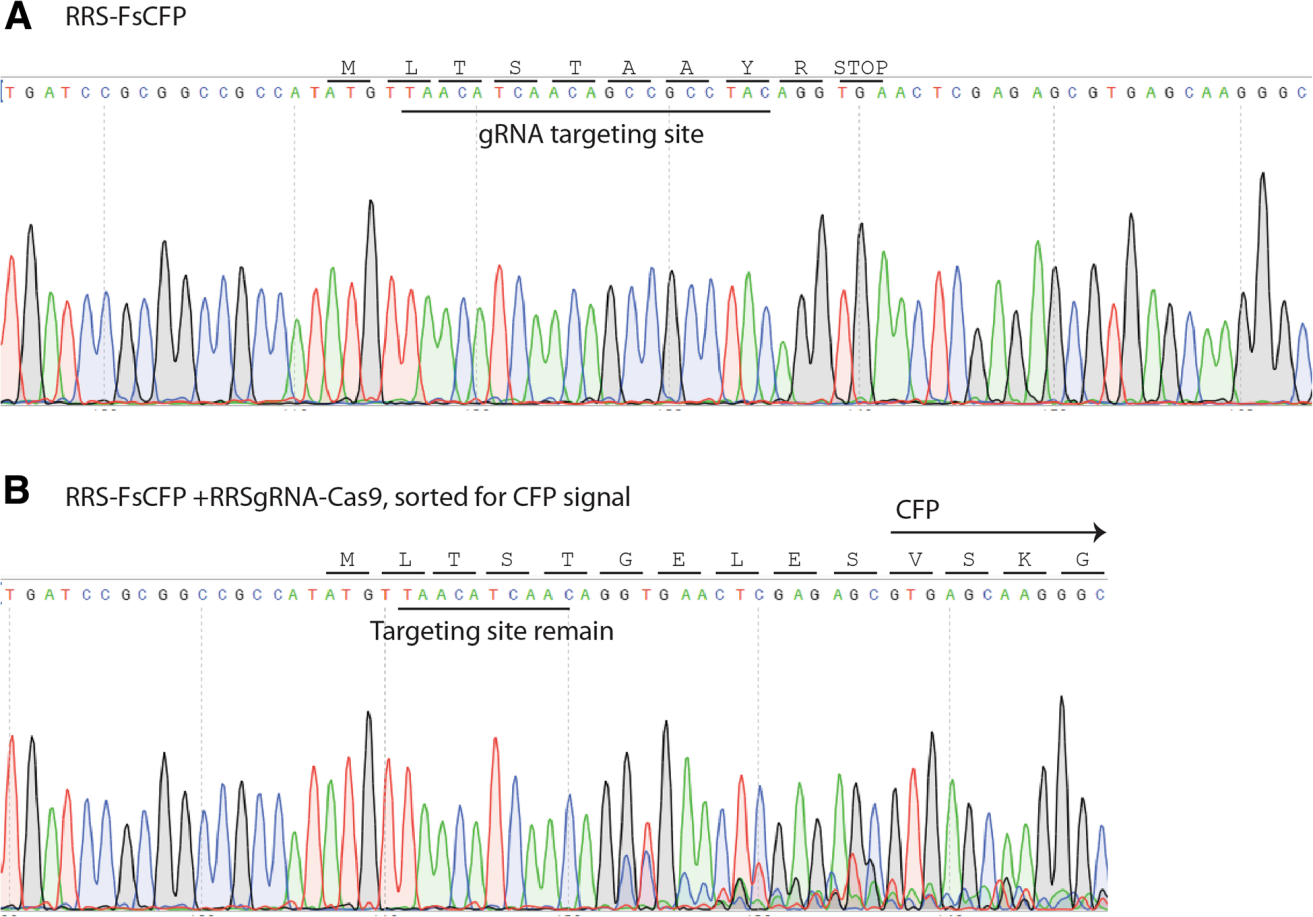
DNA sequence of genomic regions containing the FsCFP with RRS targeting sequence in cells with or without gRNA-Cas9. HEK193T cells were stably transduced with the FsCFP reporter having a target sequence derived from Chinese hamster RRS gene. The genomic region around the target sequence was amplied by a nested PCR strategy and analyzed with Sanger sequencing. **a** A representative chromatogram of the senquencing result for cells carrying only the reporter is shown. The target site for gRNA and the translation result from the start codon (ATG) was indiated. **b** A representative sequencing chromatogram for CFP-positive cells sorted by flowcytometry, which carry the reporter, wildtype gRNA and Cas9. The remainent of the target site is indicated, which appear to undergo a deletion event after the genome editing event. The translated sequence from start codon (ATG) is indicated, which is expected to generate a full-length functional CFP (indicated by the arrow)

To validate that the CFP-positive cells detected by the flow cytometer were genuine and were generated by an in/del event, we collected the CFP-positive cells generated from the RRS-targeting gRNA mentioned above to examine their fluorescence under microscope. We found that these cells exhibited genuine CFP fluorescence in addition to green and red fluorescence (Fig. 4). To further validate that the in/del events are in the expected location of the target sequence, genomic DNA was extracted from these CFP-positive cells and sequenced with Sanger method. Sequencing results showed that, in the RRSgRNA-containing cells that were enriched by CFP signals, frame-shift in/del events were detected in the gRNA-targeted region, resulting in the coding sequence of CFP in-frame for expression from the ATG starting codon (Fig. 5). Therefore, the CFP signal detected by our reporter is genuine and reported true in/del events.

## Discussion

We have demonstrated that an in/del-activated frame-shift fluorescence protein can be used as a highly sensitive tool to detect genome editing events. This reporter is quantitative in nature, extremely simple in design and operation, and in principle can be used with different cell lines in vitro or in vivo. In this study we used the CRISPR- Cas9 technique as a demonstration to evaluate the performance of this reporter. However, in principle, it can be applied to any genome editing system as long as a DSB and NHEJ are expected from the editing. Thus, this reporter can be used to evaluate the efficiency of different genome editing enzymes and can be easily adapted for high-throughput screenings. In addition, this tool can also be used for measuring and comparing the efficiency of different versions of targeting factors (such as gRNAs for CRISPR technique, or the transcription activator-like effectors for TALEN).

The newly developed FsCFP has significant advantages over other currently available tools for measuring genome editing efficiency. Compared to the Surveyor endonuclease assay, the FsCFP is much more sensitive and can effectively detect low-efficiency editing in a population of cells. In addition, unlike the Surveyor assay, the FsCFP can be used in vivo, which will greatly facilitate high-throughput screening of new genome editing enzymes. While genomic editing can also be detected by next-generation sequencing (NGS) or even by Sanger sequencing (with the help of computational algorithms such as TIDE and ICE [23, 24]), such sequencing methods are not fit for high-throughput work and are often very expensive. Also, while the detection limits are ~ 1% for methods based on Sanger sequencing and 0.1% for those based on NGS, the resolution of the frame-shift fluorescence protein is only limited by the fluorescence detection device. In the case of flow cytometer, it can easily reach a resolution better than one per million and with a much shorter turn-around time and less demand for labor. Compared to the methods based on the inactivation of GFP or other proteins, the frame-shift fluorescence protein requires a genuine in/del event to generate a positive signal, can distinguish gene editing from gene silencing, and is less likely to generate a false positive. Furthermore, in this new method, the positive cells can be conveniently identified and isolated by flow cytometry or other techniques. The positive cells can then be used for examination or validation of the in/del event. They can also be used to isolate the editing components for identification, which will be extremely helpful for people who are performing screenings of mutant libraries to obtain better genome editing tools. Finally, compared to the “traffic light reporter”, which also correlates active fluorescence to genome editing events, the FsCFP appears to have a much higher sensitivity towards NHEJ (20% *v.s*. ~ 2% in reported percentage [19, 20]. The availability of the cloning sites to swap target sequence and the use of mCherryFP as marker also make this reporter much more attractive to people for routine usages. All these features make the FsCFP much more accurate, robust, and user-friendly than the currently available methods.

## Conclusions

The insertion/deletion-activated frame-shift fluorescence protein as demonstrated in this study appears to have very low background, high sensitivity and accuracy. This reporter will facilitate the development of new genome editing tools, as well as it will also facilitate the application of existing tools such as in choosing the suitable gRNA or transcription activator-like effectors. the application of existing tools such as choosing different versions of gRNA or transcription activator-like effectors.

## Methods

### Mammalian cells and media

Human embryonic kidney cell line (HEK 293 T; clone T7, female origin) were obtained from ATCC (Manassas, VA). The Chinese Hamster ovarian (CHO) cells were a gift from Dr. Murray Deutscher (University of Miami). The mouse embryonic fibroblasts were a gift from Dr. Anna Kashina (University of Pennsylvania). These cells were grown in DMEM high glucose (Life Technologies, Cat# 11995–065) supplemented with 10% FBS (HyClone, Cat# SH30910.03), maintained at 37 °C under 5% CO2. To harvest the cells, 0.25% Trypsin solution (Life Technologies, Cat# 25200–072) were used to detach them from the culture dish/plate.

### Construction of plasmids and vectors

To construct a backbone sequence for the FsCFP-mCherryFP reporter, a nucleotide sequence containing CFP, IRES, and mCherryFP was synthesized (Genscript, NJ). This sequence is then cloned into the plasmid pQC-XIG (w497–1), which was a gift from Eric Campeau (Addgene plasmid #26826) by the cloning sites of NotI on the 5′-end and the EcoRV on the 3′-end. This resulted in the replacement of the original IRES and GFP sequences with the new CFP, IRES, and mCherryFP sequences, driven by the original CMV promoter on the plasmid. To facilitate additional cloning for the future, multiple cloning sites (MCS) were also introduced flanking the CFP and mCherryFP sequences. Particularly, a NotI site and XhoI site was used before the ATG-lacking CFP for the insertion of targeting sequence. See Additional file 1: Figure S1 for an illustration of the structure of the plasmid.

To place the targeting sequences (the WT version) in the FsCFP-mCherryFP reporter, the vector is digested with NotI and XhoI. For RRS, the targeting sequence inserted in the reporter was: TAACATCAACAGCCGCCTAC, which targets the RRS gene of Chinese hamster; for VEGF, the targeting sequence was: GGGTGGGGGGAGTTTGCTCC, which targets the VEGF-A gene in human. The targeting sequence is followed with protospacer adjacent motif (PAM) site and a premature STOP codon. The nucleotide oligos containing complementing strands of the above sequence as well as nicking nucleotides representing the digested results of NotI and XhoI were synthesized (Sigma-Aldrich) and then annealed together for ligation into the vector.

The plasmids for gRNA and Cas9 for were ordered from Vector Builder INC, with a selection marker GFP. The gRNA sequences are as below:

- RRS-gRNA: GTAACATCAACAGCCGCCTAC

(The extra G on the 5′-end was included to enhance the transcription of the U6 promoter for the gRNA, although it was not part of the targeting sequence)

- mutRRS-gRNA: GTAACATCATGAGCCGCCTAC
- VEGF-gRNA: GGGTGGGGGGAGTTTGCTCC
- mutVEGF-gRNA: GGGTGGGGCCAGTTTGCTCC

### Preparation of cell lines carrying the FsCFP-mCherryFP reporter and other fluorescence proteins

Lentiviral particles for FsCFP-mCherryFP were packaged in HEK 293 T cells using helper plasmids Delta R8.2 and VSV-G. Delta R8.2 was a gift from Didier Trono (Addgene plasmid #12263), and VSV-G was a gift from Bob Weinberg (Addgene plasmid #8454)). Transient transfection with these plasmids were done by using Polyethyleneimine (PEI) (Sigma Aldrich). Viral particles generated from these cells were harvested 24 h after initial transfection, filtered through a 0.45-uM syringe filter, supplemented with 10μg/mL polybrene, then immediately added to the target cells (HEK 293 T, CHO, or MEF) by protocols similar to described elsewhere. To minimize the possibility of multiple viral insertions per cell, the amount of virus was titrated as described elsewhere [25]. In brief, the target cells were kept in less than 75% confluence at any time during the viral induction stage. Through the course of induction, the infection rate was visually inspected under microscope to ensure fluorescence-positive cells are less than 20% at any given day. Three days following infection, successfully transduced cells containing the reporters were enriched by sorting for mCherryFP. To ensure the cells in comparison all contain the same copy numbers of reporter, the red-fluorescent cells from a single batch were used as the host cells for a new round of viral induction for viral vectors carrying the Cas9 and gRNA. As a further note to potential users, if red-fluorescent cells from different batches must be used for comparison, quantitate PCR should be used to ensure similar copy numbers of reporters exist in these cells, similar as done in our previous publications [25].

Preparation of the cells that only express GFP or mCherryFP was performed similar as described elsewhere [25].

### Sanger sequencing of genomic DNA

For Sanger sequencing of the gRNA targeting region on the FsCFP-mCherryFP reporter integrated in the cell, genomic DNA was extracted from cells with Cyclo-Prep Genomic DNA isolation kit (Amresco) and proteinase K (New England Biolabs).

A nested-PCR strategy was used to generate the nucleotide for sequencing, with these primers:

- 1st round forward primer targeting CMV promoter: AGAGCTCGTTTAGTGAACCGTC
- 1st round reverse primer targeting IRES: GACGGCAATATGGTGGAAAATAACATATAGACAAACGCACACCGG
- 2nd round for .ward primer targeting the border between the CMV promoter and the cloning sites: GAGCTCGTTTAGTGAACCGTCAGATCGCCTGGAGACGCCATCCACG
- 2nd round revers primer targeting CFP TAGTTGCCGTCGTCCTTGAAGAAGATGGTGCGCTCCTGGACGTAGCC

All PCR reactions were performed with high-fidelity DNA polymerase Herculase (Agilent Technologies). The PCR was allowed to proceed in either a Veriti Thermo Cycler (Applied Biosystems) or a T100 Thermo Cycler (BioRad).

Resulting PCR products with expected length were then submitted for Sanger sequencing with the primer targeting CFP in commercial resources such as Eurofins Genomics (Louisville, KY).

### Microscopy

Optical and fluorescent imaging of cells was performed on a Zeiss Observer equipped with a series of objectives and Zen Pro software.

### Flow cytometry analysis and fluorescence-activated cell sorting (FACS)

Flow cytometry analysis or sorting was performed on a BD Aria-IIu flow cytometer in Sylvester Comprehensive Cancer Center (SCCC) at the University of Miami. Cell fragments or aggregates were excluded based on size using side scatter and forward scatter. The remaining cells were measured for fluorescence in PE-Texas red, FITC, and CFP channels.

### Analysis of flow cytometry data

The analysis of all flow data was performed using FACSDiva v6.1.3 and FCS Express 6. The figures were generated with FCS Express 6. To set the thresholds for detecting cells with CFP fluorescence, cells expressing either only GFP, only mCherryFP, or both GFP and mCherryFP, were used as negative controls. All samples in an experiment are analyzed with the same criteria and all experimental groups were normalized to the same control.

## Additional file

Additional file 1: **Figure S1-S5.** Supplemental Figures and corresponding legends (DOCX 1627 kb)

## Data Availability

Data sharing is not applicable to this article as no data libraries were generated or analyzed during the current study. The plasmid DNA containing the frame-shift fluorescence protein, generated in this study, will be deposited in public accessible depositories including Addgene. The communication author will also accommodate requests of relevant materials.

## Abbreviations

Cas: CRISPR-associated genes
CFP: Cerulean fluorescence protein
CHO: Chinese Hamster ovary
CRISPR: Clustered regularly interspaced short palindromic repeats
DSB: DNA double stranded break
FsCFP: Frame-shift CFP
GFP: Green fluorescence protein
gRNA: guide RNA
HDR: Homology-directed repair
HEK 293 T: Human embryonic kidney 293 T
In/del: Insertion/deletion
IRES: Internal ribosome entry site
MEF: Mouse embryonic fibroblasts
NHEJ: Non-homologous end joining
NS: Non-specific
ORF: Open reading frame
RRS: Arginyl-tRNA synthase
TALEN: Transcription activator-like effector-based nucleases
VEGF: Vascular endothelial growth factor
ZFN: Zinc finger nucleases (ZFNs)
