# Supplementary material for: Insertion/deletion-activated frame-shift fluorescence protein is a sensitive reporter for genomic DNA editing
Source: BMC Genomics. 2019 Jul 24;20:609. doi: 10.1186/s12864-019-5963-z (PMC6657097; doi:10.1186/s12864-019-5963-z)
Supplement: Supplementary file 1 — Figure S1-S5. Supplemental Figures and corresponding legends (DOCX 1627 kb) [file 12864_2019_5963_MOESM1_ESM.docx]

**Supplemental Figures and legends**

**
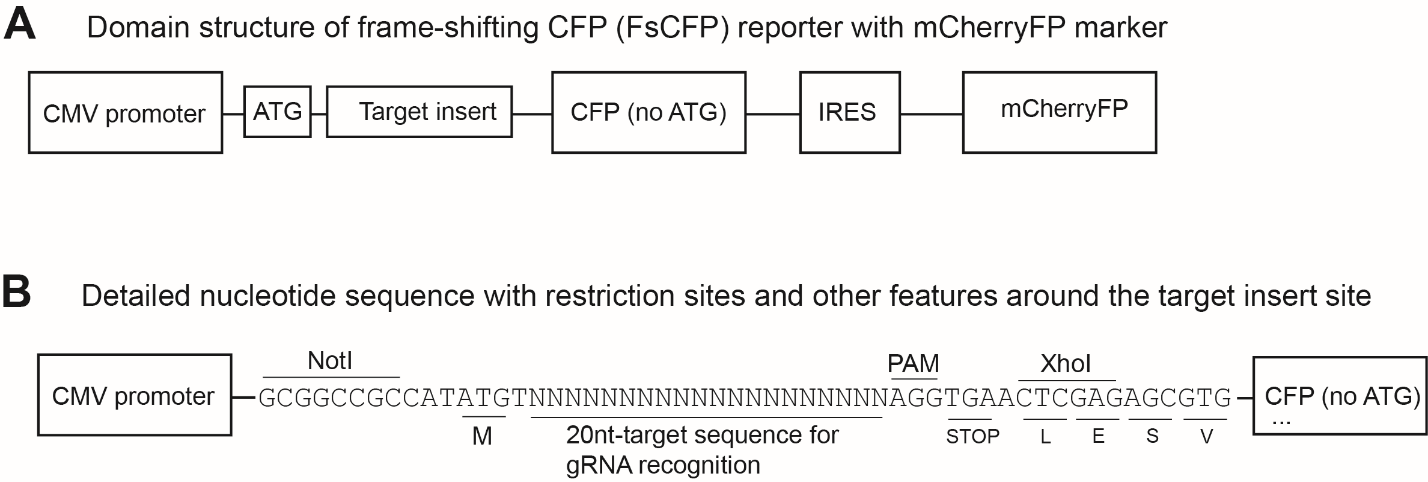
**

**Supplemental Figure S1. Related to Figure 1.** (A) The gene domain structures of the FsCFP reporter with mCherryFP trace marker, which are driven under a CMV promoter but seperated with an internal ribosome entry site (IRES) for independent translations. A nucleotide sequence for the target site is inserted between the start codon (ATG) and the rest of the coding sequence of a CFP protein (without start codon). (B) The detailed nucleotide sequence around the insert site described in A, which was cloned between a NotI and a XhoI restriction site. The position of the 20-nucleotide (nt) gRNA-matching site and the 3nt-protospacer adjacent motif (PAM) sequence for Cas9 binding are indicated. In this demonstrated example, two extra nucleotides and a premature STOP codon are also included in the inserted sequence. Together with the 20nt-gRNA-target sequence, they are expected to prevent the translation of CFP.

**
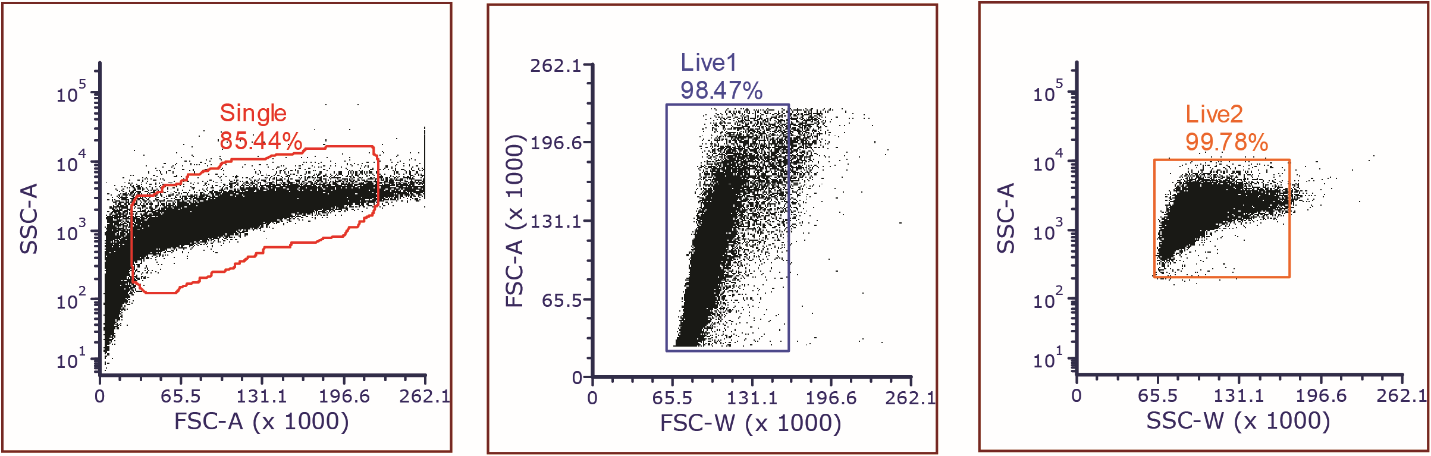
**

**Supplemental Figure S2. Related to Figure 2.**  Forward and side scattering were used to exclude cell fragments or crumps of HEK293T cells for the analysis as shown in Figure 2.

**
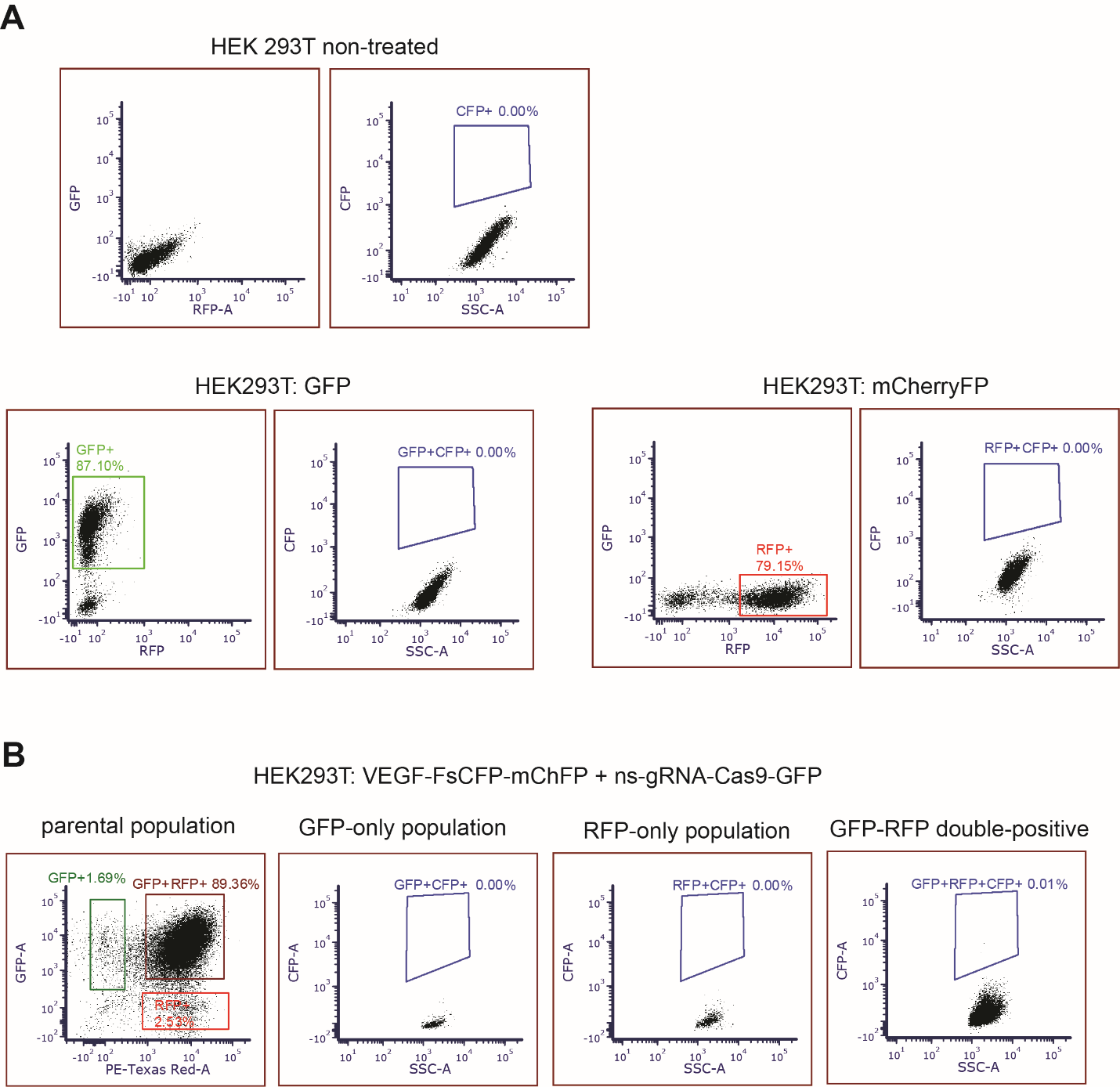
**

**Supplemental Figure S3. Related to Figure 2.** (A)The threshold for dection of the GFP and RFP/mCherryFP signals were established with HEK293T cells that were either not treated, or stably transduced with only GFP or only mCherryFP. In all these cases, no CFP signals were detected. (B) Similar to Figure 2C, except that the gates were also moved to cover the populations that only exhibit GFP or RFP signals. Compared to the GFP+RFP double-positive cells, the cells with only GFP or RFP signal contain minor numbers of CFP-positive events.


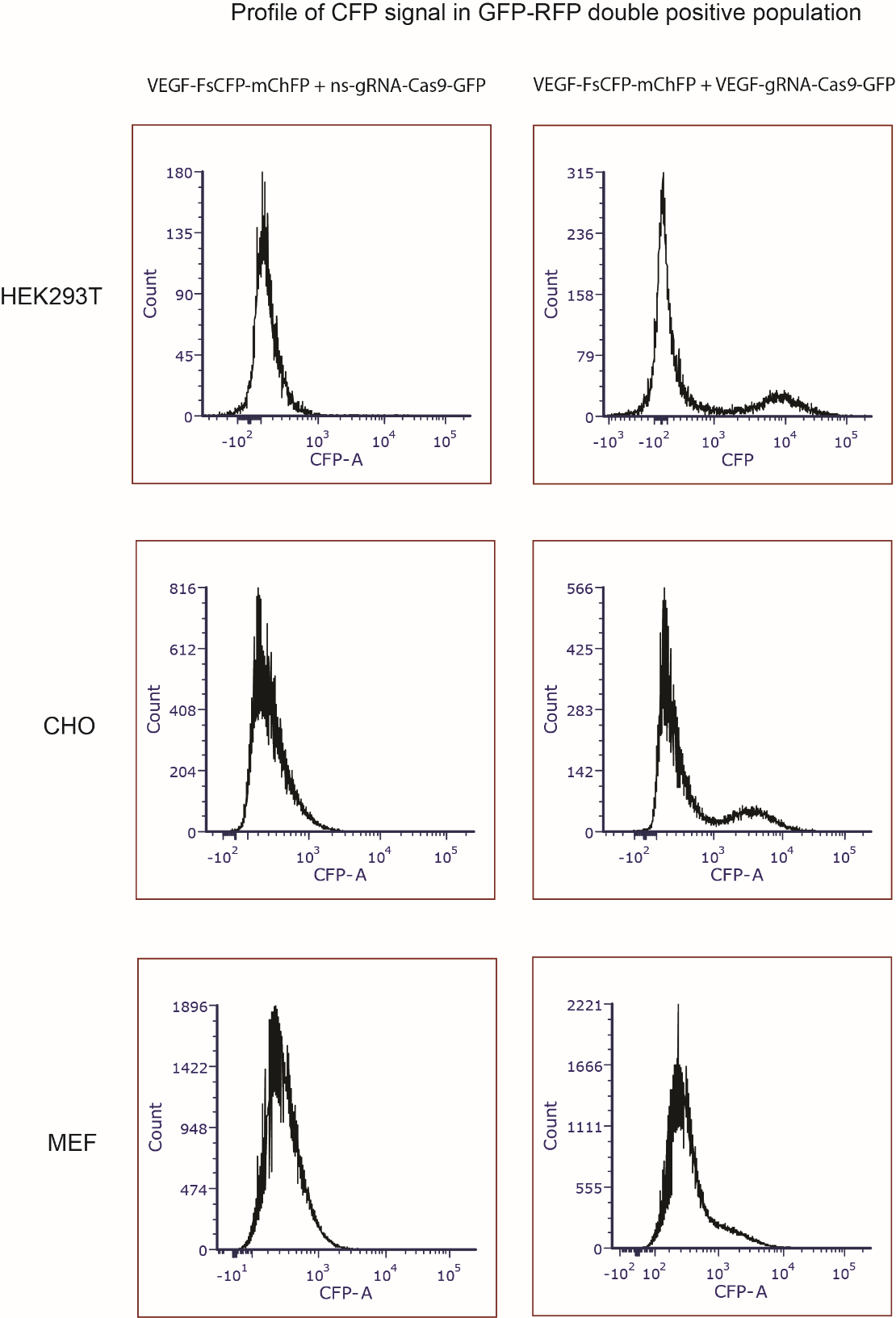


**Supplemental Figure S4. Related to Figure 2 and Figure 3.** HEK293T, CHO and MEF cells that were co-induced with an FsCFP reporter (trace marker mCherryFP) with target sequence derived from hVEGF, and Cas9 with different gRNA (WT or ns) against the hVEGF sequence (trace marker GFP). The typical distributions of CFP intensity of GFP-RFP double positive populations from these cells are shown in 1-D maps to demonstrate the difference in the CFP signals resulted from a correct gRNA and a non-specific gRNA.

**
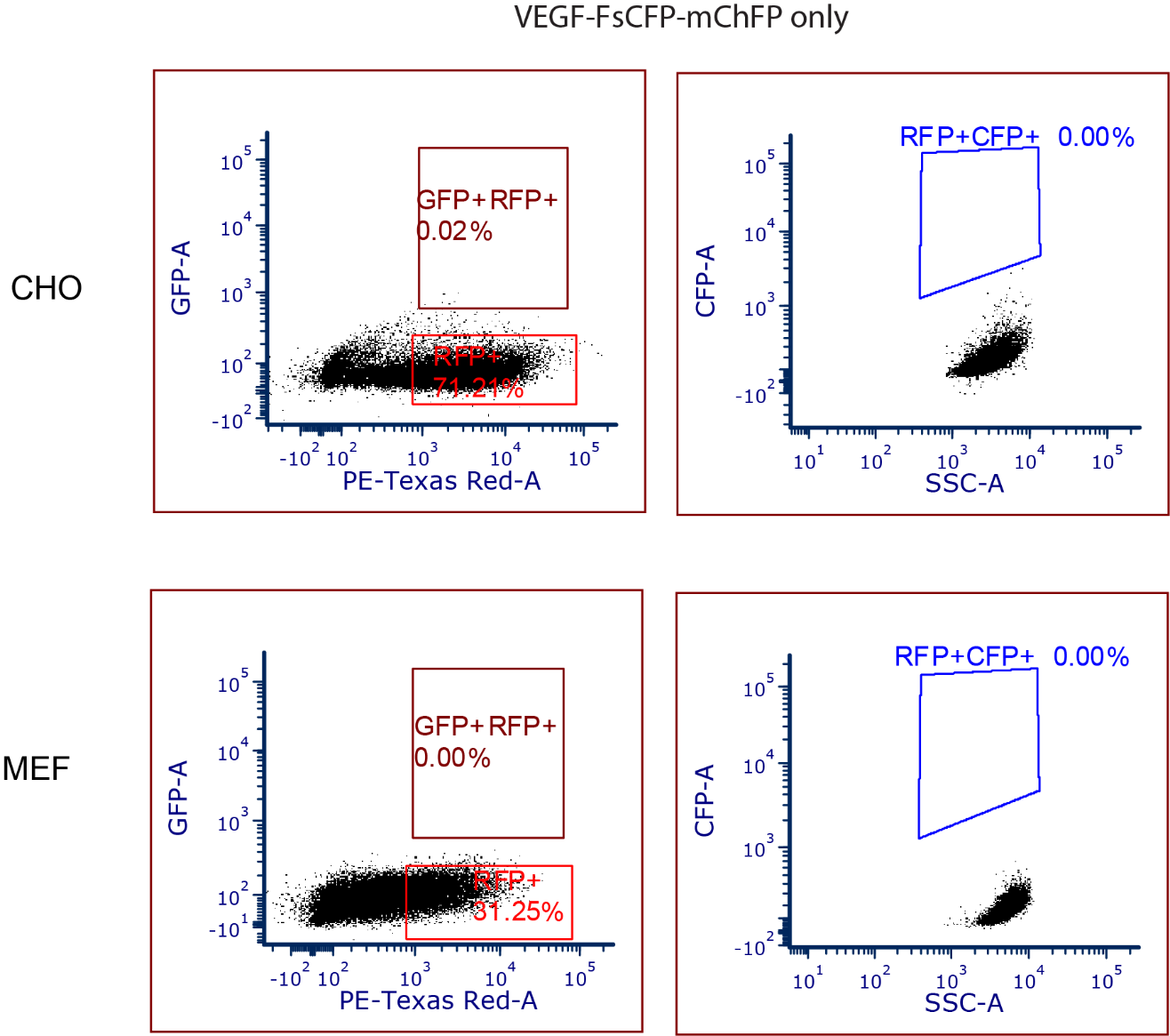
**

**Supplemental Figure S5. Related to Figure 3.** CHO and MEF cells that were virally induced with an FsCFP reporter (trace marker mCherryFP) did not show any significant CFP signals above the detection thresholds as used in Fig 3.
